# Ecological and Phylogenetic Relationships Shape the Peripheral Olfactory Systems of Highly Specialized Gall Midges (Cecidomiiydae)

**DOI:** 10.3389/fphys.2018.00323

**Published:** 2018-04-03

**Authors:** Béla P. Molnár, Tina Boddum, Sharon R. Hill, Bill S. Hansson, Ylva Hillbur, Göran Birgersson

**Affiliations:** ^1^Department of Plant Protection Biology, Swedish University of Agricultural Sciences, Alnarp, Sweden; ^2^Centre for Agricultural Research, Plant Protection Institute, Hungarian Academy of Sciences, Budapest, Hungary; ^3^Department of Evolutionary Neuroethology, Max Planck Institute for Chemical Ecology, Jena, Germany; ^4^General Directorate, International Institute for Tropical Agriculture, Ibadan, Nigeria

**Keywords:** cecidomyiidae, electrophysiology, host plant volatiles, phylogeny, specificity, COI, ef1α, 12S

## Abstract

Insects use sensitive olfactory systems to detect relevant host volatiles and avoid unsuitable hosts in a complex environmental odor landscape. Insects with short lifespans, such as gall midges (Diptera: Cecidomyiidae), are under strong selection pressure to detect and locate suitable hosts for their offspring in a short period of time. Ephemeral gall midges constitute excellent models for investigating the role of olfaction in host choice, host shift, and speciation. Midges mate near their site of emergence and females migrate in order to locate hosts for oviposition, thus females are expected to be more responsive to olfactory cues emitted by the host compared to males. In this study, we explored the correlation between host choice and the function of the peripheral olfactory system in 12 species of gall midges, including species with close phylogenetic relationships that use widely different host plants and more distantly related gall midge species that use similar hosts. We tested the antennal responses of males and females of the 12 species to a blend of 45 known insect attractants using coupled gas chromatographic-electroantennographic detection. When the species-specific response profiles of the gall midges were compared to a newly generated molecular-based phylogeny, we found they responded to the compounds in a sex- and species-specific manner. We found the physiological response profiles of species that use annual host plants, and thus have to locate their host every season, are similar for species with similar hosts despite large phylogenetic distances. In addition, we found closely related species with perennial hosts demonstrated odor response profiles that were consistent with their phylogenetic history. The ecology of the gall midges affects the tuning of the peripheral olfactory system, which in turn demonstrates a correlation between olfaction and speciation in the context of host use.

## Introduction

Olfaction evokes the most basic, often instinctive, reactions such as memory, hunger, attraction, and revulsion. Changes in the perception of the chemical world can alter the ability of individuals to survive and enable them to explore new niches or to avoid old ones—creating the classic conditions for speciation. In insects, behaviors essential for the fitness of an individual, such as mate and habitat choice, are driven to a large extent by olfaction (Hansson and Stensmyr, 2011). Thus, the relatively simple insect olfactory system is a good model to investigate speciation in the context of host preference.

Insects and plants have a long shared history (Jermy, 1984; Schoonhoven et al., 2005) with their interactions constantly modified by natural selection. The gall midge family (Cecidomyiidae: Diptera) has an ancient origin with a fossil known from the Jurassic Period. However, the gall midges expanded greatly on flowering plants during the Cretaceous Period (Gagné, 2004). There are currently more than 6,000 described gall midge species, of which approximately 80% are closely associated with flowering plants (Gagné, 2004, 2010). Of all insects, gall-inducing species are among the most host-specialized. Most gall midges are either monophagous or oligophagous (Gagné, 2004), only inducing galls on a single or a few host plant species (Carneiro et al., 2009).

The Cecidomyiidae have a high rate of speciation compared to other dipterans (Harris and Foster, 1999; Hall et al., 2012). Abrahamson et al. (1994) suggest that this rapid speciation is accelerated by two types of host-associated adaptations: host shift speciation, which is the result of a shift between two unrelated host plant species; and radiation, which is rapid speciation on a single host (Price, 2005).

Adult gall midge behavior is primarily driven by olfactory cues (Hall et al., 2012). Females use olfactory cues to attract mates by emitting a species-specific sex pheromone, and to locate suitable host plants for oviposition (Hall et al., 2012). As such, Harris and Foster (1999) hypothesized that gall midge females are more responsive to plant odors than males, as gall midge mating is associated with the site of emergence and in cases where mating is not associated with the host, females have to migrate to a suitable host for oviposition. Female host choice is crucial for larval growth and survival, as neonate larvae are small and generally unable to migrate between host plants (Gagné, 2004). Depending on the life history of the gall midge, different selection pressures may act on the olfactory system. Midges associated with annual hosts may have to migrate to locate dispersed hosts, thus employing long range olfactory cues to seek, locate, and identify a suitable host. Whereas, midges associated with perennial hosts emerge in the vicinity of host plants and may rely on short-range cues to locate and identify a suitable host.

Dipteran peripheral olfactory systems appear to be encoded in a manner consistent with the ecology of individual species (Wang et al., 2010; Bohbot and Dickens, 2012). Large-scale functional studies of the peripheral olfactory system of fruit flies (Hallem and Carlson, 2004; McBride, 2007) and mosquitoes (Carey et al., 2010; Wang et al., 2010) demonstrate that insect olfactory receptor repertoires can interact with a large range of compounds, but that the peripheral system is encoded in a way that is consistent with their ecology. For example, an insect that recently shifted to a new host does not yet have an olfactory system specifically tuned to that new host (Olsson et al., 2006a,b). However, over evolutionary time, expression of receptors that respond to the new host odors may increase and/or tuning may sharpen. Thereby, insects that have a long-term association with a host, display host-specific adaptations in their olfactory system (Stensmyr et al., 2003).

Due to the rapid speciation of gall midges, we expect that closely related gall midge species, regardless of host plant, will exhibit similar olfactory response patterns. The difference between one host smelling “right” and one host smelling “wrong,” might be due to the presence or absence of a single compound, or the variation in the ratio between compounds in the odor blend (Bruce et al., 2005; Bruce and Pickett, 2011). In other words, the physiochemical odor space (Carey et al., 2010) of closely related gall midges is predicted to mirror their phylogeny. However, if the shift to a new host plant applies strong selection pressure on the peripheral olfactory system of the midge, rapid adaptation may result. We expect that such changes would be out of proportion with the common rate of midge adaptation, and thus the chemical odor space would correlate with the host plant rather than with gall midge phylogeny.

In this study, we use combined gas chromatography and electroantennographic detection (GC-EAD) recordings to analyze the antennal responses of 12 gall midge species to a wide range of host plant-related volatiles. Electrophysiological techniques measuring peripheral olfactory neuronal response, such as GC-EAD and electroantennography (EAG), have traditionally been used for sex pheromone identification. More recently EAG and GC-EAD have been used to identify plant volatiles that may play an important role in insect-plant interactions (Blight et al., 1997; Honda et al., 1998). Recent studies propose that EAG responses to plant volatiles can be species-specific and that there is a correlation between antennal response spectra, host specificity and preference breadth (Ngumbi et al., 2010). EAGs are generally believed to measure summarized electrical potentials created by simultaneously activated olfactory sensory neurons (OSNs) lying in series on the antenna (Schneider, 1962, 1969; Kaissling, 1971; Roelofs, 1984) and the amplitude response potential is directly proportional to antennal length (Kaissling, 1971; Nagai, 1983). Since EAG amplitude is subject to change depending on connection strength, insect vitality as well as the position of the electrode (Olsson and Hansson, 2013), the EAG response should in general be treated as a qualitative, rather than quantitative indicator of olfactory perception (Olsson and Hansson, 2013). Based on this, we recorded responses evoked by certain chemical compounds as a yes or no, instead of measuring EAG amplitudes. By comparing the olfactory response of phylogenetically close and distantly related gall midges the following questions are addressed:

1. Do distantly related gall midges associated with the same host plant use the same or a similar set of odors to identify it? Do closely related species that have different host plant requirements, respond to odors in common with the different plants?
2. Are olfactory responses sex dependent? We expect that female gall midges are generally more sensitive therefore respond better to plant volatiles. As gall midge mating takes place at the site of emergence, there is no selection pressure on the male olfactory system; therefore, we do not expect male olfactory responses to match host plant, but to reflect the midge phylogeny.
3. Is the life history of gall midges reflected in their EAG response pattern? As midges associated with annual plants might have to locate the host each season—in some cases over a great distance—we expect the response to closely match the odor profile of the host. In contrast, there is less selection pressure on the olfactory system of midges associated with perennial hosts, as they will emerge near their host. Therefore, we expect that their response will mirror their phylogeny.

## Materials and methods

### Insects

The 12 species were included in the electrophysiological assays: *Dasineura napi* (brassica pod midge), *Dasineura gleditchiae* (honeylocust gall midge), *Obolodiplosis robiniae* (black locust gall midge), *Resseliella theobaldi* (raspberry cane midge), *Mikiola fagi* (beech gall midge), *Monarthropalpus flavus* (boxwood leafminer), *Dasineura pyri* (pear leafcurling midge), *Contarinia sorghicola* (sorghum midge), *Dasineura mali* (apple leaf curling midge), *Mayetiola destructor* (Hessian fly), *Aphidoletes aphidimyza* (aphid predator midge), and *Contarinia nasturtii* (swede midge).

The majority of the species used in the study were field collected. *Dasineura napi, D. gleditichiae, O. robiniae*, and *R. theobaldi* were collected in Southern Sweden. *Monarthropalpus flavus* and *D. pyri* were collected near Budapest, Hungary. *Contarinia sorghicola* was collected near Acona, Italy and *D. mali* in Lincoln, New Zealand. For all these species, infested plant material containing larvae was placed in ventilated acrylic glass cages (50 × 50 × 50 cm) with 5 cm of potting soil in a climatic chamber (22 ± 1°C, 65 ± 5% RH, 16 h light: 8 h dark photoperiod) until adult midges emerged. Beech leaves infested with *M. fagi* were collected in the southernmost province of Sweden (Skåne) in late autumn (Oct.-Nov.) and placed in acrylic glass cages with a 10 cm layer of potting soil and stored in a climatic chamber (5 ± 1°C, 85 ± 5% RH) for 3 months in order for the larvae to complete diapause. After 3 months cages were placed in the same climatic chamber described above until the midges emerged.

Adult midges or pupae of *M. destructor, A. aphidimyza*, and *C. nasturtii* originated from laboratory cultures. Pupae of *M. destructor* were received from a laboratory culture at North Dakota State University, Fargo, ND, US. The pupae were placed on damp filter paper in an acrylic cage in climatic chamber (conditions as above) until the adults emerged. Pupae of *A. aphidimyza* were bought from Koppert B. V. Postbus, The Netherlands and placed in an acrylic cage with 5 cm of potting soil then placed in a climatic chamber (conditions as above). The *C. nasturtii* originated from a laboratory culture reared at Swedish University of Agricultural Sciences, Alnarp, Sweden (Boddum et al., 2009). Additionally, four species, *Contarinia tritici* (lemon wheat blossom midge), *Contarinia pisi* (pea midge), *Sitodiplosis mosellana* (orange wheat blossom midge), *Cecidomyia piniinopis* (gouty pitch midge), were field collected, but due to low population numbers specimens were included only in the molecular study.

### Molecular biology

**Figure 1:**
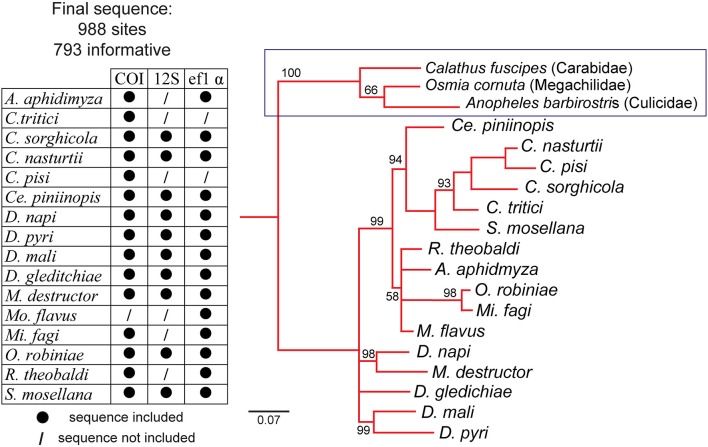
Neighbor-joining tree of 12 gall midge species based on the cytochrome oxidase subunit I (COI) gene of mitochondrial DNA (mtDNA), small ribosomal RNA gene (12S), and a part of elongation factor 1-α (ef1 α). *Calathus fuscipes* (Carabidae)*, Osmia cornuta* (Megachilidae)*, Anopheles barbirostris* (Culicidae) were used as outgroup species. Sequences in the chart marked with “•” are included in the present phylogenetic reconstruction while sequences marked with “/” are not included.

Approximately 10 specimens of each species were pooled and genomic DNA was extracted using a DNeasy Blood & Tissue Kit (QIAGEN, Sweden). Regions of three genes were amplified by polymerase chain reaction (PCR): mitochondrial cytochrome c oxidase subunit I (COI) (forward primer: GGA GGA TTT GGA AAT TGA TTA GTT CC, reverse primer: CCC GGT AAA ATT AAA ATA TAA ACT TC-3′; predicted size of the fragments 590 bp) (Simon et al., 1994), elongation factor 1-α (ef1α) (forward primer: AAA ATG CCAT GGT TCA AAG G, reverse primer: CGA AAT TTG ACC TGGA TGG T; predicted size of the fragments 568 bp) (Joy and Crespi, 2007), and 12S small ribosomal gene (12S) (forward primer: TAC TAG TTA CGA CTT AT, reverse primer: AAA CTA GGA TTA GAT ACC C; predicted size of the fragments 430 bp) (Dorchin et al., 2004). The fragments were purified using a QIAquick Gel extraction kit (QIAGEN). Both strands of the PCR products were directly sequenced by Sanger sequencing (MWG Eurofins, Germany). COI sequences for *A. aphidimyza, M. fagi, R. theobaldi C. tritici*, and *C. pisi* were obtained from GenBank (accession numbers AB028157.1, AB162848.1, AB506024.1, AY485383.1, and AY485382.1, respectively). For some species, the concentration of amplified DNA was too low for direct sequencing, and are therefore not included in the present phylogenetic reconstruction (Figure 1). Gene sequences are available from the figshare database: https://doi.org/10.6084/m9.figshare.3808173.v1.

### Phylogenetic analysis

Prior to gene sequence alignment, the best fit model of sequence evolution was determined using JModel test (Posada and Crandall, 1998). Sequences were aligned with Muscle multiple sequence alignment in SeaView version 4 (Gouy et al., 2010). Bayesian phylogenetic analysis was conducted in MrBayes (version 3.2) (Huelsenbeck and Ronquist, 2001) with 1,000 bootstrapped replications.

### Synthetic plant compounds

**Figure 2:**
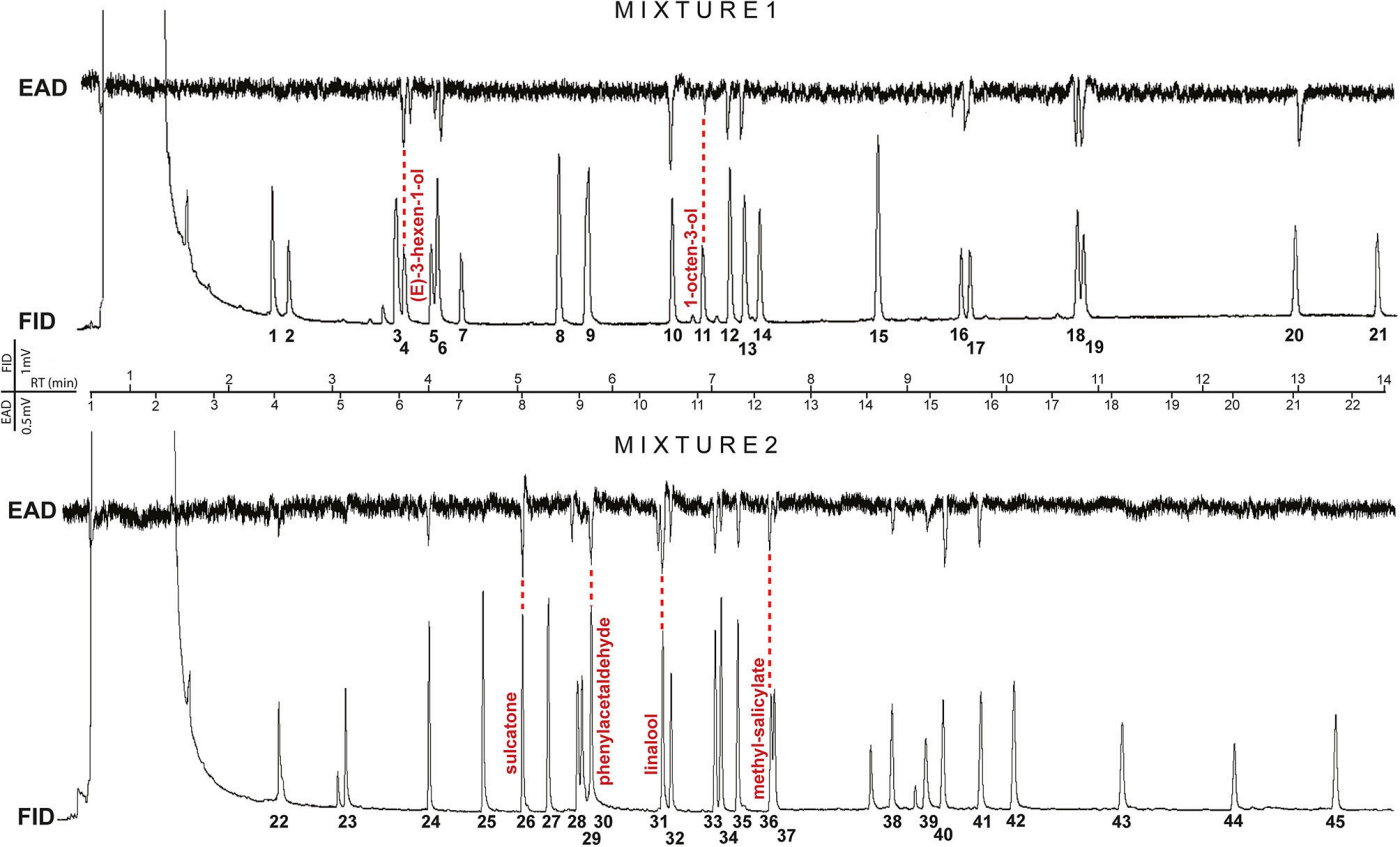
Representative GC-EAD traces of gall midge odorant receptor neurons (ORNs) response profiles to plant volatiles. Upper trace shows male *Dasineura pyri* responses to compounds 1–21 (mixture 1; Table 1), and the lower trace shows female *Dasineura mali* responses to compounds 22–45 (mixture 2; for compounds see Table 1). Compounds with most abundant response % are named and corresponding antennal responses are indicated by a red, dashed line.

Chemical compounds (Table 1) were selected to represent the most relevant chemical groups from host plants in relation to the gall midges. The blend was combined to contain a wide variety of plant volatiles, such as alcohols, aldehydes, aromatic compounds, monoterpenes, sesquiterpenes, and isothyocianates (ITC), known to be involved in insect attraction with special focus on known gall midge attractants (Ruther, 2000; Birkett et al., 2004; Anfora et al., 2005; Hopkins et al., 2009). A total of 45 individual compounds were tested in two mixtures (Figure 2). The compounds were sorted into two mixtures based on their retention indices to ensure appropriate separation by the gas chromatograph (GC) and to avoid co-elution for electroantennographic detection (EAD). The concentration of each compound in both mixtures was 10 ng/μl dissolved in redistilled *n*-hexane (≥98.0%, Sigma-Aldrich).

**Table 1:** Plant volatile compounds tested in the GC-EAD analysis on 12 gall midge species. “#” refers to the numbers of the peak in Figure 2.

**#**	**Chemical compound**	**Purity (%)**	**CAS number**	**Source**
1	Ethyl propionate	99	105-37-3	VWR International
2	2-Hexanone	96	591-78-6	Sigma-Aldrich
3	Hexanal	98	66-25-1	Sigma-Aldrich
4	(E)-3-hexen-1-ol	98	928-97-2	Sigma-Aldrich
5	(Z)-3-hexen-1-ol	98	928-96-1	Sigma-Aldrich
6	(E)-2-hexen-1-ol	97	928-95-0	Sigma-Aldrich
7	(Z)-2-hexen-1-ol	95	928-94-9	Acros Organics
8	Allyl isothiocyanate	98	1957.06.07	Sigma-Aldrich
9	α-Pinene	98	80-56-8	Acros Organics
10	Camphene	95	79-92-5	Sigma-Aldrich
11	1-Octen-3-ol	98	3391-86-4	Acros Organics
12	Myrcene	95	123-35-3	Sigma-Aldrich
13	n-Butyl-isothiocyanate	98	592-82-5	Sigma-Aldrich
14	(Z)-3-hexenyl acetate	98	3681-71-8	Sigma-Aldrich
15	Hexyl acetate	99	142-92-7	Sigma-Aldrich
16	Terpinolene	97	586-62-9	Sigma-Aldrich
17	Limonene (-)	95+	138-86-3	Sigma-Aldrich
18	Limonene (+)	97+	5989-27-5	VWR International
19	Hexyl-butyrate	98	2639-63-6	Sigma-Aldrich
20	(E)-2-hexenyl-butyrate	97	53398-83-7	Sigma-Aldrich
21	Geranylacetone	98	3796-70-1	Sigma-Aldrich
22	(Z)-3-hexenal	50	6789-80-6	Sigma-Aldrich
23	(E)-2-hexenal	98	85761-70-2	Sigma-Aldrich
24	Isobutyl-isobutyrate	98	97-85-8	Sigma-Aldrich
25	Benzaldehyde	98+	100-52-7	Sigma-Aldrich
26	Sulcatone	99+	409-02-9	Sigma-Aldrich
27	3-Carene	95+	13466-78-9	Sigma-Aldrich
28	2-Ethyl-1-hexanol	99	104-76-7	Sigma-Aldrich
29	Benzyl-alcohol	99+	100-51-6	Sigma-Aldrich
30	Phenylacetaldehyde	95+	122-78-1	Sigma-Aldrich
31	Linalool ±	97	78-70-6	Sigma-Aldrich
32	(Z)-3-hexenyl propionate	97+	33467-74-2	Sigma-Aldrich
33	(Z)-3-hexenyl-isobutyrate	98+	41519-23-7	Sigma-Aldrich
34	Hexyl-isobutyrate	98+	2349.07.07	Sigma-Aldrich
35	Benzyl-acetate	99+	140-11-4	Sigma-Aldrich
36	Methyl-salicylate	98	119-36-8	Sigma-Aldrich
37	3-Methylthiopropyl isothiocyanate	98+	505-79-3	Sigma-Aldrich
38	Hexyl tiglate	97+	16930-96-4	Sigma-Aldrich
39	Benzyl isothocyanate	98+	622-78-6	Sigma-Aldrich
40	(Z)-3-hexenyl hexanoate	98	31501-11-8	Sigma-Aldrich
41	β-Caryophyllene	98,5	87-44-5	Sigma-Aldrich
42	α-Humulene	97	6753-98-6	Acros Organics
43	(Z)-3-hexenyl benzoate	98+	25152-85-6	Sigma-Aldrich
44	Isopropyl-myristate	98	110-27-0	Sigma-Aldrich
45	1-Hexadecanol	99+	36653-82-4	Sigma-Aldrich

### Electrophysiology

GC-EAD (Arn et al., 1975), was performed using an Agilent 6890N GC (Agilent Technologies Inc., Santa Clara, CA, USA), equipped with a HP-5 coated fused silica capillary column (30 m × 0.25 mm; *df* = 0.25 μm, J&W Scientific, Folsom, CA, USA) in 30 s splitless mode. The oven temperature was held at 30°C for 2 min, then increased at 8°C min^−1^ to 220°C and held for 2 min. The injector temperature was set at 220°C. Hydrogen was the mobile phase at constant flow rate of 45 cm s^−1^. At the GC effluent, nitrogen (4 psi) was added as a make-up gas and split equally in a low dead volume four-way splitter (Gerstel 3D/2, Gerstel, Mülheim, Germany). Two identical pieces of deactivated fused silica capillary column (100 cm × 0.25 mm) were connected to the four-way splitter, one led to the flame ionization detector (FID) (280°C) and the other to the heated EAD port (220°C; Gerstel ODP2 transfer line). The EAD capillary effluent was delivered to the antennal preparation in a stream of charcoal-filtered and humidified air in a glass tube (ID 8 × 150 mm; airflow 500 ml/min).

To generate stable GC-EAD recordings (*n* = 10 per sex per species), the head of a newly emerged gall midge was excised and inserted into glass capillary (ID 1.17 mm, Syntech, Hilversum, the Netherlands) filled with Ringer's solution (Beadle and Ephrussi, 1936) and attached to the reference silver/silver chloride electrode held in a micromanipulator. The tips of both intact antennae were simultaneously inserted into the recording glass electrode shaped to provide a narrow opening and also filled with Ringer's solution. Before and after each EAD recording with a mixture, the quality of the antennal preparation was tested using a Pasteur pipette stimulus with filter paper cartridge loaded with 10 μl from the other mixture (10 ng μl^−1^).

The antennal signal was amplified (10x), converted to a digital signal by a high input impedance DC amplifier interface (IDAC-2, Syntech, Germany) and recorded simultaneously with the FID signal on a computer using the GC-EAD 2010 software (version 1.2.3, Syntech). For every recording, a new antennal preparation was used and 2 μl of the synthetic blend (10 ng μl^−1^) was injected to the GC.

Electrophysiological responses to the synthetic blend were analyzed by visualizing the recordings (GC-EAD 2010) and scoring the responses to the single compounds as “present” or “absent.” The percentage responding to each compound was later calculated for both sexes of each species. A heat plot of the responses was generated manually using a conditional formatting application with graded color scale in Microsoft Excel 2011 (version 14.3.9). Three step color-coding (black: low or no response, through blue to red: high response percentage) was used to represent the response percentage as a variable in a hierarchy. The comparisons between male and female responses were made using a Wald Chi-square test (binary probit analyzes, generalized linear model; SPSS 20, version 18). The neighbor joining cluster analysis was carried out to compare the proximity of the GC-EAD responses to the panel of compounds among the different gall midge species. PAST 2.16 software (Hammer et al., 2001) with the Bray-Curtis similarity index was used to generate the cluster analyzes. Metadata used in the above analyses is available from the figshare database: https://doi.org/10.6084/m9.figshare.3484880.v1.

## Results

### General trends

**Figure 3:**
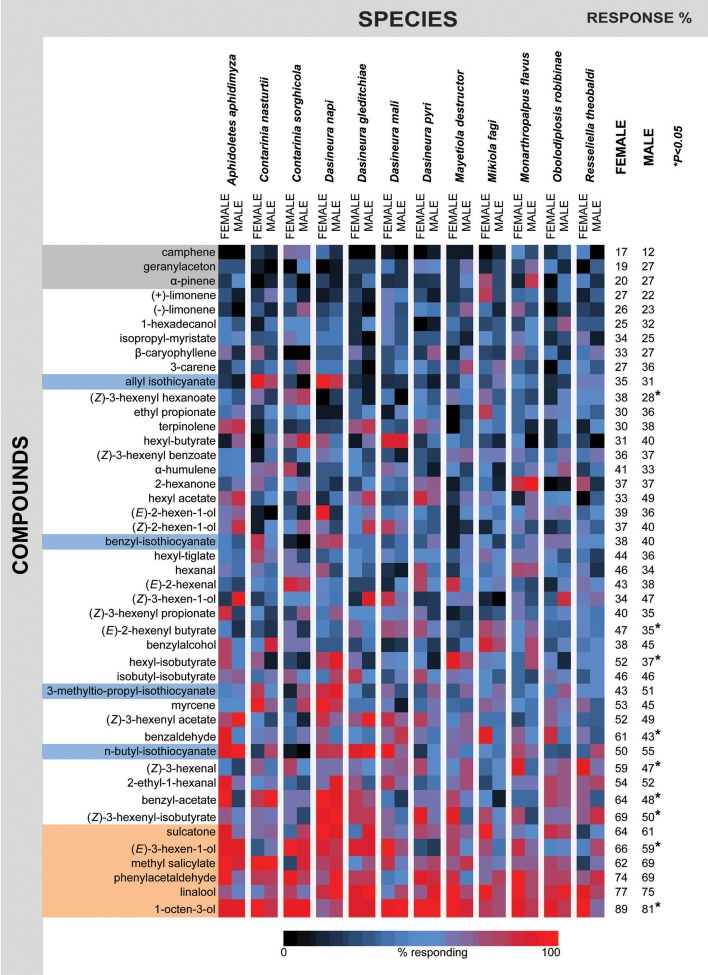
Heat plot of the summarized GC-EAD response profiles of 12 gall midge species. Graphical representation of summarized ORN response profile of 10 individuals of each sex from12 gall midge species to 45 plant volatile compounds. Response intensity is color-coded according to the continuous color scale on the bottom, the compounds are color highlighted according to the following annotation: blue: Crucifer specific compounds, orange: eliciting strong responses in many tested midges, gray: only few responding midges. Significant differences between male and female responses to certain compounds indicated by asterisk (^*^) on the right in the figure (*P* < 0.05; Wald Chi-square test, binary probit analyzes, generalized linear model).

The phylogeny among the 12 midge species tested is based on the DNA sequence similarities among three loci, COI, ef1α, 12S (Figure 1). Every individual midge tested responded to 7–20 compounds in the panel (Figure 2). More than 50% of the gall midges, independent of sex and species, responded to six of the compounds: 1-octen-3-ol (89% females, 81% males), linalool (77% females, 75% females), phenylacetaldehyde (74% females, 69% males), methyl salicylate (62% females, 69% males), (*E*)-3-hexen-1-ol (66% females, 59% males), and sulcatone (6-methyl-5-hepten-2-one; 64% females, 61% males; Figure 3, compounds highlighted in orange). Less than 30% of the midges responded to three of the odors: camphene (17% females, 12% males), geranylacetone (19% females, 27% males), and α-pinene (20% females, 27% males; Figure 3, compounds highlighted in gray).

Within each species, only a few compounds elicited antennal responses in all individuals (Figure 3, red squares) or in no individuals (black squares). Instead, there was high within-species variation in response profiles, with some, but not all individuals capable of detecting the compounds (Figure 3, blue squares). This response pattern resulted in species-specific response profiles that are used in the following analyses.

### Differences between sexes

Overall, both males and females responded to an array of the tested compounds (Figure 3). For most compounds, there was no difference when the responses of all females were compared to the response of all males (Figure 3, right side panel). Nine compounds evoked responses in significantly more females than males: (*Z*)-3-hexenyl-hexanoate, (*E*)-2-hexenyl-butyrate, hexyl-isobutyrate, benzaldehyde, (*Z*)-3-hexenal, benzyl acetate, (*Z*)-3-hexenyl-isobutyrate, (*E*)-3-hexen-1-ol, and 1-octen-3-ol (Figure 3).

### The glucosinolates—crucifer specific compounds

The two crucifer specific gall midges—*C. nasturtii* and *D. napi*—were the only species that responded to all four types of crucifer specific glucosinolate degradation products; the isothiocyanates: allyl isothiocyanate, benzyl-isothiocyanate, 3-methylthio-propyl-isothiocyanate, and n-butyl-isothiocyanate (marked with light blue in Figure 3). There was a sex-dependent difference in the way the two species responded. In *D. napi*, males and females both responded to all crucifer compounds. For *C. nasturtii*, only females responded to benzyl-isothiocyanate and 3-methylthio-propyl-isothiocyanate. Both sexes responded to allyl isothicyanate, while only the male *C. nasturtii* responded significantly to n-butyl-isothiocyanate. Females of *C. nasturtii* and *C. sorghicola* showed the lowest response to that compound. Half of all females and half of all males, including *A. aphidimyza, D. mali, D. gleditchiae, M. flavus*, male *R. theobaldi*, and female *M. destructor* responded to n-butyl isothiocyanate.

### Host plant associated differences

**Figure 4:**
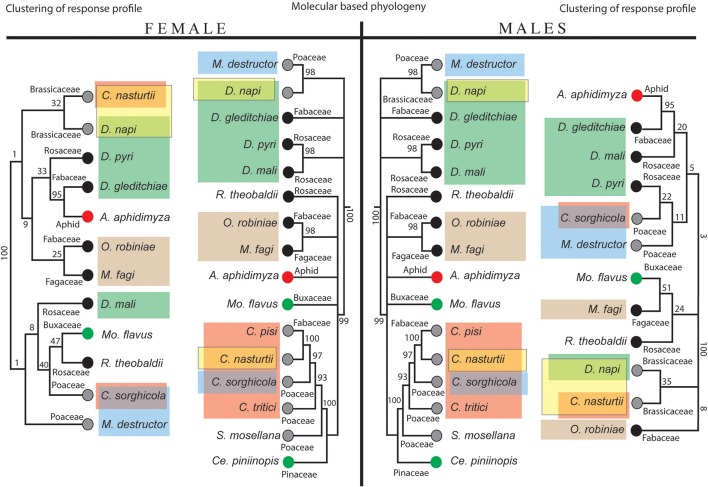
Neighbor-joining trees of species-specific antennal response profiles (female on the left, male on the right) in comparison with molecular-based phylogeny (middle part of the figure). Gray dots indicate annual host plant, black dots perennial host plant; green circle for evergreen hosts and the red dots indicate entomophagous feeding habit. Species are highlighted according to the following annotation; green: *Dasineura* species; blue: species utilize Poaceae hosts; yellow: Brassica specialists; brown: indicates close phylogenetic relationship of *O. robiniea* (Fabaceae) and *M. fagi* (Fagaceae) however males showed distinct host preference.

A comparison between the neighbor-joining trees of the species-specific response profiles of females (Figure 4, left panel) and males (Figure 4, right panel) with the molecular-based phylogeny of the gall midges (Figure 4, middle panel) demonstrate that neither the female nor the male response trees were in complete agreement with the phylogenetic tree. However, there is a group of closely related *Dasineura* species (male: *D. napi, D. pyri, D. gleditchiae*; female: *D. gleditchiae, D. mali, D. pyri*), where the response profiles mirror the phylogeny (Figure 4, green box). Thus, there is a difference in how the response of *D. mali* matches the phylogenetic tree between the sexes. The response of male *D. mali* clusters with the other *Dasineura* species, whereas the response profile of female *D. mali* clearly separates from the other *Dasineura* species.

Interestingly, the response profile for *D. napi* (both males and females) is similar to the profile of the other crucifer specialist, *C. nasturtii* (Figure 4, yellow box). The response profiles of the two species cluster together, in contrast to the large phylogenetic distance between the two species. A similar pattern is found for males and females of the two species associated with grasses, *M. destructor* (wheat) and *C. sorghicola* (sorghum) where despite the large phylogenetic distance between them, their antennal responses group together (Figure 4, light blue box). For one set of species however, the opposite pattern was observed. *O. robiniae* and *M. fagi* cluster in the phylogenetic tree (Figure 4, brown box). For the females, the response profiles also cluster. However, the response profiles of the males are distant in the response tree.

## Discussion

Here, we functionally described the peripheral olfactory system of 12 species of male and female gall midges by GC-EAD screening to an array of plant volatiles and compared their antennal response profiles in the context of their phylogenetic and host plant relationships. The current understanding of the phylogenetic relationship among gall midges is primarily based on morphological traits. However, there are aspects of the classical systematic analyses that are unsettled and problematic (Dorchin et al., 2009). For this study, we produced a new molecular-based phylogeny for the involved species. The resulting phylogenetic tree appears to be consistent with the morphology-based phylogeny, except for *M. destructor* (Gagné, 2004).

A few compounds elicited responses in almost all individuals for each species tested. These compounds are possibly of high biological relevance as most are general plant volatiles widely distributed within the plant kingdom. For instance 1-octen-3-ol, (*Z*)-3-hexenyl acetate, and 2-ethyl-1-hexanol, which are known host cues for the orange wheat blossom midge (Birkett et al., 2004), elicited responses in many species with a variety of hosts, supporting the ratio-specific hypothesis of host plant recognition (Bruce et al., 2005). We fully realize that electroantennogram responses to plant volatiles do not necessarily mean they are integrated by the CNS to elicit a behavioral response (Del Socorro et al., 2010a,b), but this is a good first step toward identifying these behaviourally relevant stimuli.

In addition to the general responses noted above between species, we also found a large difference in response to many of the tested compounds within-species. Despite gall midge host specificity, midge preference depends on host availability; a mated female will oviposit on a less preferred host in the absence of a preferred host (Boddum, unpublished). Female choice is important for gall midge evolution because mating is associated with the site of oviposition (Harris and Foster, 1999). Interestingly, since the majority of the midge species in this study were wild caught, the large variation in the response profiles to many of the tested compounds demonstrates pronounced within-population variation in the olfactory system. This variation can be the basis for rapid adaptation to new hosts and an explanation for the comparatively high rate of speciation in the family. For such a rapid change in the tuning of the peripheral olfactory system to accommodate a host switch, pre-existing variability in the response to odors among the individuals within the population, such as we have demonstrated, would be expected.

### Comparison of phylogenetic and functional relationships

Within the small number of midge species tested, we found instances where olfactory response profiles matched evolutionary relationships and where olfactory response profiles matched host plant use (Figure 3). For instance, the responses of *C. nasturtii* and *C. sorghicola* did not correlate with their phylogeny, but rather their host plant use. *Contarinia* is one of the largest gall midge genera, with at least 300 species (Yukawa et al., 2005). Compared to other gall midges, *Contarinia* species are less closely associated with their hosts than other gall inducing insects and most of them live freely in flower heads or gregariously in leaf rolls or leaf fold galls (Gagné, 2004). Furthermore, there are very few examples of non-specialized, polyphagous gall midges, but one of them is a *Contarinia* species, *C. maculipennis* Felt, that infests at least eight plant families (Uechi et al., 2011; Tokuda, 2012). The fact that *Contarinia* species can be found on a wide range of plant families, and not associated with any specific host plant genus (Yukawa et al., 2005), indicates that host plant shifts occur more frequently in *Contarinia* species than in species more closely associated with their host. This ecological trait seems to be mirrored in their olfactory system in which the response profiles of the two tested *Contarinia* species are not clustered in the neighbor-joining response profile tree as they are in the phylogenetic tree. The observed odor response pattern of the peripheral olfactory system demonstrates that the ecology of *Contarinia* gall midges shapes the function of their olfactory systems. Due to the importance of olfactory cues in host finding (Birkett et al., 2004), the olfactory system is subject to diversifying selection when the midges enter a new niche (e.g., during a host shifts and subsequent speciation events). McBride (2007) described the co-occurrence of host specialization and receptor evolution in *Drosophila sechellia*, demonstrating that olfactory receptor genes experience increased selection pressure during a dramatic ecological change.

Host use shaping the olfactory system of certain clusters of gall midges is further underpinned by the similar response profiles of the two Brassicaceae specialists, *C. nasturii* and *D. napi*. Despite large phylogenetic distance between the two species, only these species responded to all the crucifer specific isothiocyanates. The glucosinolate-myrosinase defense system present in Brassicaceae plants produces toxic secondary metabolites such as isothiocyanates following cell damage which are involved in plant defense (Fahey et al., 2001). The distinct difference in response patterns of the Brassica specialists may be explained by disruptive selection following the first encounter with the unique chemical defenses of the plants (Städler and Reifenrath, 2009). The first encounter with this plant type may have applied strong selection pressure on the peripheral olfactory system of the midge, and rapid evolutionary adaptation may have resulted. After colonization of the new host, the two distantly related Brassica specialist gall midge species continue to respond to the host specific compounds, and stabilizing selection may have maintained this host specific adaptation.

Our GC-EAD data provided further examples of olfactory systems adapted to host preference. *Aphidoletes aphidimyza* is the only zoophagous midge in our study; its larvae prey on almost all true aphids (van Lenteren et al., 2002). Still, adults responded to several plant compounds and to herbivore induced plant volatiles (HIPV), indicating that female midges may use plant volatiles to localize the aphid colony. Benzaldehyde and methyl salicylate elicited responses in almost all tested specimens of *A. aphidimyza*, especially in females. Interestingly, release of either benzaldehyde or methyl salicylate with the conspecific sex pheromone, synergize, and increase trap catches of the bird cherry-oat aphid (*Rhopalosiphum padi*) and damson-hop aphid (*Phorodon humuli*) (Pope et al., 2007). Compounds such as linalool, sulcatone, and (*E*)-3-hexenyl acetate also elicited high responses in both sexes of *A. aphidimyza*. These compounds are known as part of an effective synthetic kairomone blend of the polyphagous *Aphis fabea*, the black bean aphid (Webster et al., 2008). Linalool and methyl salicylate are also known HIPV (Röse and Tumlinson, 2004) and thus the presence of these compounds in a plant volatile profile may attract predators and parasitoids (Dicke, 2009). Potentially these HIPV compounds could also mediate the host-finding behavior of the aphid predator midge as attractants.

The preferred oviposition site of the female *M. fagi* is the dorsal side of the European beech (*Fagus sylvatica*) leaf. Females can distinguish the dorsal and ventral side of the leaves using plant volatiles. Volatiles emitted from dorsal side contain sulcatone, (*E*)-3-hexen-1-ol and methyl salicylate at much higher concentrations than the ventral side of beech leaves (Molnár, unpublished). This preliminary study correlates well with our current results, in which sulcatone and (*E*)-3-hexen-1-ol evoked a response in a high percentage of tested *M. fagi* females. This suggests that those volatiles and/or their ratio may play an important role in female oviposition site choice.

We also identified patterns where the olfactory tuning reflected the evolutionary relationship of the midges, not host use. One such example is the group of *Dasineura* species. Interestingly, most of the tested *Dasineura* species are associated with perennial hosts. Midges associated with perennial hosts will emerge close to their host, thus there is less pressure on the olfactory system to locate a host, compared to species associated with annual hosts. Due to the rapid speciation of gall midges, their olfactory system might not have host-specific neurons. Instead, it is likely that they distinguish a host from a non-host by the presence or absence of a single compound or, more likely, by variation in the ratio between compounds in the odor blend.

The response of female *D. mali* is distinctly different from the response of the other female *Dasineura*. The two species, *D. mali* and *D. pyri* are morphologically similar in traits commonly used to distinguish gall midges (Galanihe and Harris, 1997) and they were not regarded as two well-defined species until olfactory experiments demonstrated that *D. mali*, the apple leaf curling midge, prefers apple foliage over pear foliage (Galanihe and Harris, 1997), while *D. pyri* exclusively feed on pear leaves. Our results show that females of the two species respond differently to some of the tested compounds. The response difference was not due to host specific compounds—as was the case for the crucifer species—instead they responded differently to common compounds found in many flowering plants. Evolutionary radiation within the same host plant species, or between similar host species (in this case apple and pear) allows gall midge species to explore a new niche, but does not require extensive adaptations as does the challenging shift to a different host plant (Joy and Crespi, 2007). Radiation as a speciation mechanism for *D. mali* and *D. pyri* is also supported by our data. Despite the close phylogenetic relationship, the electrophysiological response pattern of female *D. mali* and *D. pyri* is different and separate from each other. However, the difference is caused by response to common plant compounds, and thus do not require excessive adaptations of the olfactory system. It is interesting, that the responses of male *D. mali* and *D. pyri* still are well clustered together. This indicates that stress on the olfactory system associated with a host shift, primarily act on the females. However, as the response profile of most other males matches host use, we assume that the male olfactory system will follow the female's response profiles.

### Sex dependent differences

Contrary to what we hypothesized, the male and female antennal response profile trees were similar with a few exceptions. It might be possible that males may have a higher probability of finding a female near a host plant, so they should also be tuned to host plant.

However it has been reported that only the female gall midges migrate to the host for oviposition (Readshaw, 1966; Thygesen, 1966; Summers, 1975) and we therefore expected females generally respond to a larger number of plant compounds. However, as males and females within a species responded similarly to host plant compounds, their peripheral olfactory systems appear to be shaped by the same processes and not only by sex-specific processes. This finding questions the assumption that only mated females migrate to the host and is further supported by Samietz et al. (2012) who, in a field experiment, caught male midges in a host plant field some distance away from the site of emergence.

Our data show that males are responsive to host plant volatiles and they may also be attracted to the host plant. This indicates that mating in gall midges does not exclusively take place at site of emergence, but might also be associated with the host.

## Author contributions

BM and TB: Participated equally in the design of the study, collected gall midge species, selected and designed mixtures of VOCs, carried out experiments and molecular biology lab work, and performed statistical analyses; SH: Mentored the molecular biology work and wrote the manuscript together with BM and TB; GB: Mentored the chemical analysis part of the study and participated in the design of the mixtures; YH and BH: Financed, mentored, participated in the design of the study, and helped to finalize the manuscript. All authors read and approved the final version of the manuscript.

### Conflict of interest statement

The authors declare that the research was conducted in the absence of any commercial or financial relationships that could be construed as a potential conflict of interest.
